# Hepatic overexpression of methionine sulfoxide reductase A reduces atherosclerosis in apolipoprotein E-deficient mice[S]

**DOI:** 10.1194/jlr.M058776

**Published:** 2015-10

**Authors:** Yan-Yong Xu, Fen Du, Bing Meng, Guang-Hui Xie, Jia Cao, Daping Fan, Hong Yu

**Affiliations:** * Department of Biochemistry and Molecular Biology Wuhan University School of Basic Medical Sciences, Wuhan, China; † Hubei Provincial Key Laboratory of Developmentally Originated Disease, Wuhan University School of Basic Medical Sciences, Wuhan, China; § Department of Cell Biology and Anatomy, School of Medicine, University of South Carolina, Columbia, SC

**Keywords:** antioxidants, cholesterol efflux, cholesterol elimination, lipoprotein metabolism, inflammation, lipoprotein receptors, paraoxonase-1

## Abstract

Methionine sulfoxide reductase A (MsrA), a specific enzyme that converts methionine-*S*-sulfoxide to methionine, plays an important role in the regulation of protein function and the maintenance of redox homeostasis. In this study, we examined the impact of hepatic MsrA overexpression on lipid metabolism and atherosclerosis in apoE-deficient (apoE^−/−^) mice. In vitro study showed that in HepG2 cells, lentivirus-mediated human MsrA (hMsrA) overexpression upregulated the expression levels of several key lipoprotein-metabolism-related genes such as liver X receptor α, scavenger receptor class B type I, and ABCA1. ApoE^−/−^ mice were intravenously injected with lentivirus to achieve high-level hMsrA expression predominantly in the liver. We found that hepatic hMsrA expression significantly reduced plasma VLDL/LDL levels, improved plasma superoxide dismutase, and paraoxonase-1 activities, and decreased plasma serum amyloid A level in apoE^−/−^ mice fed a Western diet, by significantly altering the expression of several genes in the liver involving cholesterol selective uptake, conversion and excretion into bile, TG biosynthesis, and inflammation. Moreover, overexpression of hMsrA resulted in reduced hepatic steatosis and aortic atherosclerosis. These results suggest that hepatic MsrA may be an effective therapeutic target for ameliorating dyslipidemia and reducing atherosclerosis-related cardiovascular diseases.

Atherosclerosis is a multifactorial chronic inflammatory disease. Numerous genetic and environmental contributing factors have been documented, among which dyslipidemia and oxidative stress are well-known risk factors (1–3). The liver plays a critical metabolic role in regulating lipid and redox homeostasis through many processes including synthesis of cholesterol, VLDL secretion, the uptake of cholesterol from the peripheral tissues, and its excretion into bile by a process called reverse cholesterol transport (RCT). Hepatic dysfunction could aggravate the progression of atherosclerosis via deterioration of dyslipidemia and increased levels of circulating proinflammatory factors (4, 5). Therefore, manipulation of hepatic lipid metabolism and inflammation is a commonly pursued strategy to prevent atherosclerosis progression.

There is considerable evidence that cellular homeostasis and pathological states are affected by redox status. The accumulation of reactive oxygen species (ROS) can lead to oxidative modification of biomolecules and cell damage. Methionine (Met) sulfoxide formation is one of the most common posttranslational modifications of proteins when ROS levels are increased. Cellular methionine sulfoxide reductase A (MsrA), as an essential oxidoreductase, specifically catalyzes the reduction of free and protein-based methionine-*S*-sulfoxide (MetSO) to Met. It is expressed in various cell types with different levels and regulates intracellular redox status (6, 7). A number of studies have demonstrated that MsrA plays protective roles in repairing reversible protein Met oxidation; thus it protects cells from oxidative stress and contributes to the prevention of oxidation-related disease such as cataracts, Alzheimer’s disease, and ischemia/reperfusion injury (7–11). Recent studies have shown that a single polymorphism, rs10903323 G/A, in the human MsrA (hMsrA) gene is associated with an increased risk of coronary artery disease (12, 13), highlighting the importance of MsrA.

Although MsrA is expressed in hepatocytes, its impact on hepatic lipoprotein metabolism and function has not been studied (14). We hypothesized that hepatic expression of MsrA might attenuate the progression of atherosclerosis by regulating redox state and some key proteins involved in lipid metabolism and inflammation. To test this hypothesis, we used lentivirus injection to achieve high-level hepatic expression of hMsrA in apoE-deficient (apoE^−/−^) mice. We found that hepatic hMsrA expression ameliorated lipid metabolism dysfunction and reduced the progression of atherosclerosis in Western-diet-fed apoE^−/−^ mice, through altering the expression of several genes involved in lipid metabolism and inflammation in the liver. These findings provide new insights into the biological effects of MsrA in the liver and suggest that MsrA may be a promising therapeutic target for atherosclerosis.

## METHODS

### Materials and reagents

#### Mice.

ApoE^−/−^ mice (on a C57BL/6 background) were purchased from Vital River Laboratory Animal Technology Co. (China) and housed in microisolator cages in Wuhan University Animal Center. Animal care and experimental procedures were performed under the regulations of the Institutional Animal Care and the Ethics Committee for Animal Experiments of Wuhan University conforming to the Public Health Service Policy on Humane Care and Use of Laboratory Animals. Sixteen male apoE^−/−^ mice at 11 months of age were randomly divided into two groups for lentiviral injection. Starting at 2 weeks after lentiviral injection, the mice were fed with AIN76A Western diet (purchased from Beijing HFK Bioscience Co., China) for 12 weeks to accelerate the development of atherosclerosis. The composition of the diet was the following: 20% milk fat, 19.5% casein, 10% maltodextrin, 5% corn starch, 5% cellulose, and 0.15% cholesterol.

#### Generation of plasmids and production of lentiviruses.

The plasmid containing hMsrA cDNA sequence was a gift from Prof. Stefan H. Heinemann (Jena Friedrich Schiller University, Germany). To generate a lentiviral vector for hMsrA overexpression, a 639 bp cDNA fragment of hMsrA was amplified by PCR and subcloned into a bicistronic lentiviral vector PWPI containing tagged green fluorescent protein (GFP). The final hMsrA-lentiviral construct PWPI-hMsrA-GFP was verified by DNA sequencing. General procedures for lentivirus production were described previously (15). The lentivirus for overexpression of hMsrA was named Lv-MsrA-GFP, and the control lentivirus was named Lv-GFP.

#### Overexpression of hMsrA in HepG2 cells.

HepG2 hepatocellular line was purchased from Classic Specimen Culture and Storage Center at Wuhan University (Wuhan, China). The cells were cultured in high-glucose DMEM (Gibco) supplemented with 1% penicillin/streptomycin, 2 mM glutamine, and 10% FBS (Gibco) in 5% CO_2_ saturated humidity, at 37°C. Cells were subcultured at 1 × 10^5^ cells per well into 6-well culture plates. After 12 h of culture, the cells were transfected with the PWPI-hMsrA-GFP vector or the control PWPI-GFP vector using Lipofectamine 2000 (Invitrogen) according to the manufacturer’s instructions.

#### Tissue distribution of MsrA in mice.

All mice were injected with 200 µl of lentivirus containing 1.6 × 10^8^ particles of either Lv-MsrA-GFP or Lv-GFP via the retroorbital venous plexus under anesthesia with 3% isoflurane, in order to achieve high-level expression of MsrA and GFP simultaneously or GFP alone, respectively. Two weeks after lentiviral injection, the mice were fed with a Western diet for 12 weeks and then euthanized, and tissues were harvested. Livers were cyrosectioned at 5–10 µm and directly visualized for GFP fluorescence using an Olympus microscope. Expression of MsrA protein in liver and other tissues including aorta, heart, spleen, and kidney was determined by immunohistochemistry and/or Western blot analysis.

#### Plasma lipid, lipoprotein, and biochemical analyses.

Beginning at 2 weeks after lentiviral injection, blood samples were collected from mice after overnight fasting by retroorbital venous plexus puncture at 2- to 4-week intervals. Plasma was immediately separated by centrifugation at 10,000 *g* for 10 min at 4°C and then aliquoted and stored at −80°C. Total cholesterol (TC) and TG levels in fresh plasma were determined enzymatically using Cholesterol Reagent and TG GPO Reagent kits (Prodia Diagnostics, Boetzingen, Germany). HDL-cholesterol (HDL-C) levels were determined after precipitation of the apoB-containing lipoproteins using HDL-C reagent kits according to the manufacturer’s instructions (Sekisui Medical, Japan). Plasma lipoprotein profiles were determined by fast-protein liquid chromatography (FPLC) using a Superose 6 10/300 GL column (Amersham Biosciences) on an AKTA purifier (GE Healthcare) as described previously (16).

Plasma paraoxonase-1 (PON1) activity was measured as described previously (17). An enzymatic colorimetric assay was conducted to determine the activity of superoxide dismutase (SOD) using SOD reagent kits (Nanjing Jiancheng Bioengineering Institute, China). Plasma alanine aminotransferase (ALT) was determined using Mohum’s method.

#### Biochemical and histochemical analyses of the liver.

Fresh liver tissue was homogenized in PBS, and total lipids were extracted using isopropanol. The amounts of TC, free cholesterol (FC), cholesteryl ester (CE), and TG were determined as previously described (16, 18). Lipid content was normalized to liver protein content. Histochemical analysis of liver tissue was performed by staining with hematoxylin and eosin (HE) or Oil Red O (5 µm serial sections), and images were acquired using an Olympus microscope.

#### Fecal cholesterol measurement.

Mice feces were collected, and cholesterol was extracted from the dried feces using isopropanol. After evaporation under nitrogen, the extracts were solubilized in isopropanol containing 10% Triton X-100, and the TC levels were determined. Total fecal cholesterol was normalized to dry weight of the feces, and data are presented as milligrams (cholesterol) per gram (dry weight).

#### Quantification of atherosclerotic lesions.

Twelve weeks after lentiviral injection, the extent of atherosclerosis was examined using Oil Red O-stained cross sections of the aortic root (8 µm serial sections) and by en face analysis of the aorta. The quantification was performed using Image-Pro Plus 6.0 software as previously described (16, 18).

#### Quantitative real-time PCR analysis.

Total mouse liver RNA was isolated with TRIzol reagent and reverse transcribed into cDNA with reverse transcriptase (Invitrogen). The cDNA products were subjected to quantitative real-time PCR analysis using universal PCR master mix (Invitrogen). The PCR primers for amplification of mouse genes, including apoAI, low density lipoprotein receptor (LDLR), scavenger receptor class B type I (SR-BI), liver X receptor alpha (LXRα), ABCA1/G8, ACAT, cholesterol 7-α hydroxylase (CYP7A1), cholesterol 27-α hydroxylase (CYP27A1), acetyl-CoA carboxylase (ACCa), FASN, PON1, interleukin-6 (IL-6), and TNFα, are shown in supplementary Table 1. The RT-PCR program consisted of 1 cycle at 95°C for 5 min followed by 40 cycles at 95°C for 30 s, 60°C for 30 s, and 72°C for 30 s. The expression level of each gene was normalized to the level of 18S RNA.

#### Western blot analysis.

Western blot was used to measure the target protein levels in plasma, cell lysate, or tissue homogenate. The protein concentrations of cell lysate and liver homogenate were determined by the Lowry method using a DC protein assay kit (Bio-Rad), and appropriate amounts of proteins (supplementary Table 2) were loaded and separated by SDS-PAGE and transferred onto nitrocellulose membranes, and target proteins were detected by specific primary antibodies and HRP-conjugated secondary antibodies. The signal was detected using an ECL kit. The detailed information about loading amount of proteins and primary antibodies against mouse or hMsrA, apoAI, LDLR, SR-BI, LXRα, ABCA1, ABCG8, cholesteryl ester hydrolase (CEH), ACAT, PON1, serum amyloid A (SAA), GAPDH, or β-actin were also shown in supplementary Table 2.

### Statistical analysis

Data are presented as mean ± standard error of the mean. The Mann-Whitney test was used to measure the statistical differences in lesion area. The other results were analyzed using two-tailed Student *t*-test. The differences were considered significant with *P* < 0.05.

## RESULTS

### hMsrA overexpression affects expression of lipoprotein-metabolism-related genes in HepG2 cells

**Fig. 1.**
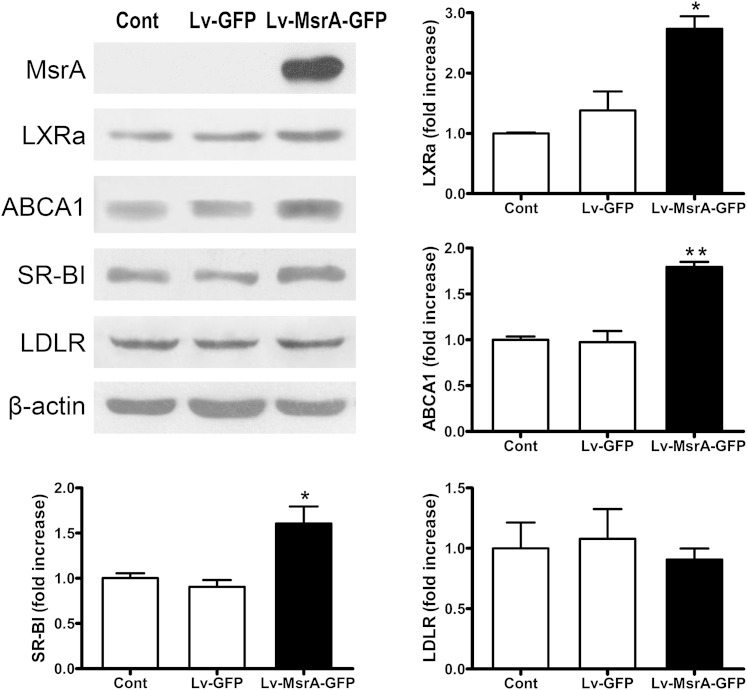
hMsrA overexpression affects protein expression levels of lipoprotein-metabolism-related genes in HepG2 cells. The cells were collected on the third day after transfection with PWPI-hMsrA-GFP vector or the control PWPI-GFP vector using Lipofectamine 2000. Total cell proteins were extracted, and expression of LXRα, ABCA1, SR-BI, and LDLR proteins was determined by Western blot analysis using anti-human LXRα, ABCA1, SR-BI, and LDLR antibodies and anti-human β-actin antibody as loading control. Relative fold increase of each target protein is shown (mean ± SD, n = 3). * *P* < 0.05 and ** *P* < 0.01.

We used lentiviral vector PWPI-GFP as the starting construct to generate a bicistronic lentiviral vector to overexpress hMsrA and GFP (PWPI-hMsrA-GFP) (supplementary Fig. 1). Next, PWPI-hMsrA-GFP plasmids were transfected into the HepG2 cells to confirm the overexpression of hMsrA and GFP (supplementary Fig. 2). We then examined the effects of hMsrA overexpression on the expression of several cholesterol-metabolism-related genes. The results showed that overexpression of hMsrA in HepG2 cells significantly increased the expression levels of LXRα, ABCA1, and SR-BI, but not that of LDLR (**Fig. 1**). The results suggested that MsrA overexpression in hepatocytes may be able to regulate cholesterol uptake and efflux through increasing the expression of the genes mentioned previously.

### Hepatic high-level expression of hMsrA in Lv-MsrA-GFP mice

**Fig. 2.**
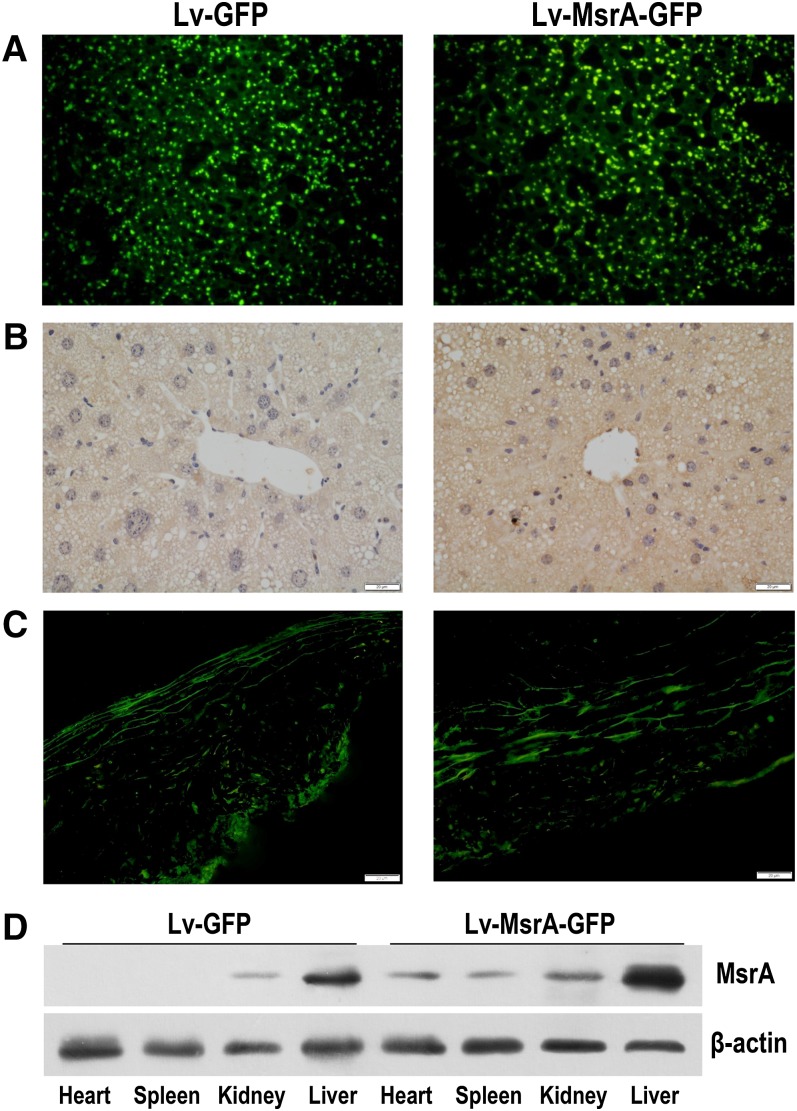
hMsrA is predominantly expressed in the liver in mice treated with lentivirus injection. Lentiviruses Lv-MsrA-GFP or Lv-GFP were intravenously injected into 11-month-old apoE^−/−^ mice. The mice were euthanized 14 weeks after injection, and tissues were harvested. A: GFP expression of liver cryosections (5 µm) in both Lv-MsrA-GFP and the control Lv-GFP mice were directly visualized under the fluorescence microscope. B, C: Expression of MsrA protein in the liver or aorta was determined by immunohistochemistry. D: MsrA levels in the liver and other tissues including heart, spleen, and kidney were detected by Western blot analysis using anti-hMsrA antibody. β-actin was detected as the loading control.

Lentiviruses Lv-MsrA-GFP or Lv-GFP were intravenously injected into 11-month-old apoE^−/−^ mice. Two weeks after the lentiviral injection, the mice were fed a Western diet until euthanized after an additional 12 weeks. Fluorescence of GFP in liver cryosections and MsrA proteins in liver or aorta cryosections was detected. The results showed that GFP or MsrA proteins were expressed in liver and aortic tissue in both control Lv-GFP-injected (Lv-GFP) mice and Lv-MsrA-GFP-injected (Lv-MsrA-GFP) mice (**Fig. 2A–C**), and the hepatic MsrA expression level in Lv-MsrA-GFP mice was significantly higher than that in Lv-GFP mice (Fig. 2B). Western blot analysis also showed that the hepatic MsrA level in Lv-MsrA-GFP mice was increased 3.4-fold when compared with that of Lv-GFP mice (Fig. 2D). Although the MsrA levels in extrahepatic tissues of Lv-MsrA-GFP mice were higher compared with those of Lv-GFP mice, MsrA expression in the liver was 5- to 9-fold higher compared with the expression levels in heart, spleen, and kidney (Fig. 2D), suggesting that intravenous injection of Lv-MsrA-GFP predominantly increased the expression of hMsrA in the liver. On the other hand, hepatic high-level expression of hMsrA did not cause in vivo toxicity, and no alterations were observed in body weight, spleen weight, or plasma ALT activity (supplementary Fig. 3).

### Plasma lipid levels and oxidative status were ameliorated in Lv-MsrA-GFP mice

**Fig. 3.**
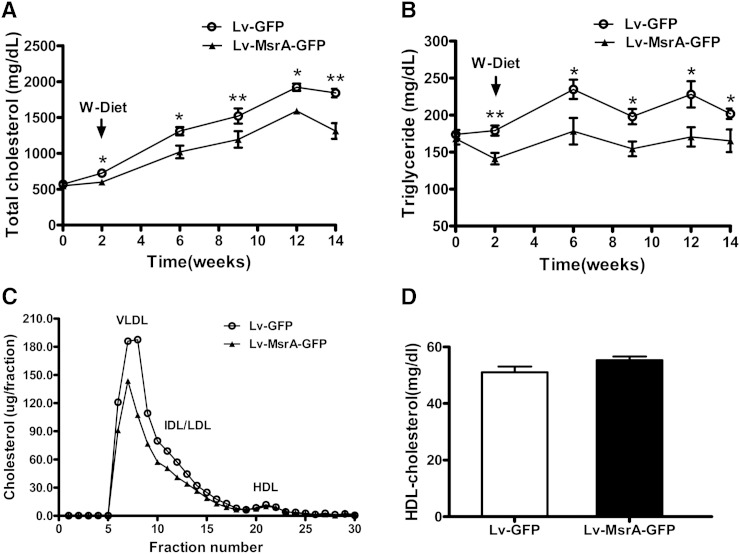
Hepatic hMsrA expression reduces plasma VLDL/LDL-cholesterol (LDL-C) levels. Lentiviruses Lv-MsrA-GFP or Lv-GFP (1.6 × 10^8^ particles in 200 µl) were intravenously injected into 11-month-old apoE^−/−^ mice. Starting at 2 weeks after the lentiviral injection, the mice were fed a Western diet for 12 weeks. A, B: TC and TG levels of fasting plasma collected at the indicated time were determined enzymatically as described in Methods. Data are presented as mean ± SD, n = 8. * *P* < 0.05 and ** *P* < 0.01. C: Plasma lipoprotein profiles in Lv-MsrA-GFP or Lv-GFP mice at 12 weeks after Western diet feeding. Lipoproteins in pooled plasma were size fractionated by FPLC using a Superose 6 10/300 GL column, and cholesterol contents within lipoprotein fractions (VLDL, LDL, and HDL) were measured. D: Plasma HDL-C level in Lv-MsrA-GFP or Lv-GFP mice were measured after precipitation of the apoB-containing lipoproteins.

Plasma lipid levels were assessed on the day of lentiviral injection and once every 2 or 3 weeks thereafter. Fasting plasma TC and TG levels and FPLC profile were measured, and the results showed that hepatic hMsrA expression reduced plasma lipid levels in apoE^−/−^ mice as early as 2 weeks after lentiviral injection, and the reduction became more significant during the 12-week period with Western diet feeding (**Fig. 3A, B**). The FPLC profiling indicated that the reduction in plasma TC and TG was due to decreases in VLDL and LDL fractions, but not in HDL fraction (Fig. 3C). Direct measurement of plasma HDL-C after depletion of apoB-containing lipoproteins also confirmed that the HDL-C level was not significantly changed (Fig. 3D).

**Fig. 4.**
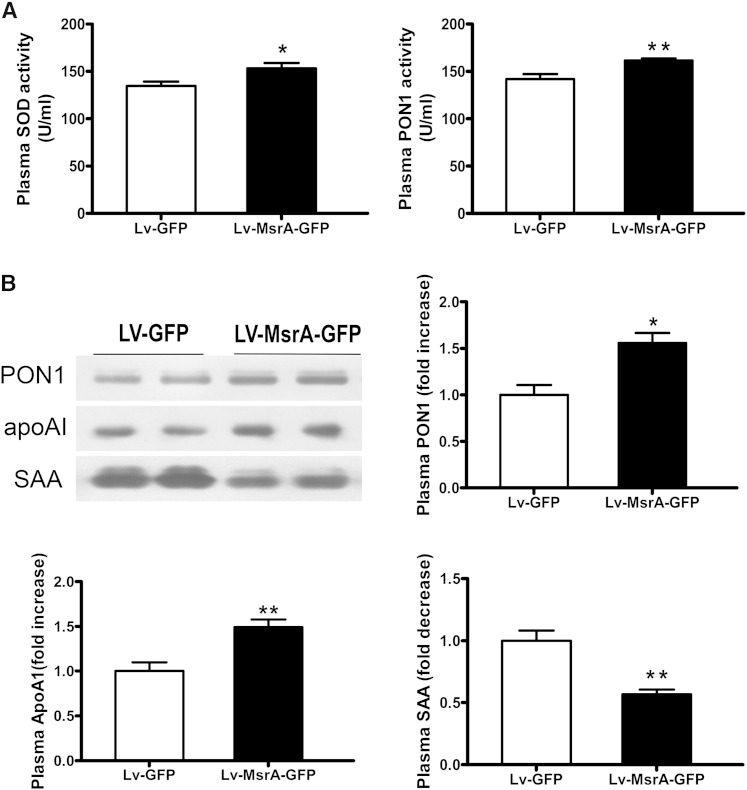
Hepatic hMsrA expression improves plasma redox state. A: The activities of antioxidative enzymes SOD and PON1 in plasma were determined by enzymatic colorimetric assay. Data are presented as mean ± SD, n = 8. * *P* < 0.05. B: Plasma protein levels of PON1, apoAI, and SAA were determined by Western blot analysis using anti-mouse PON1, apoAI, and SAA antibody. Relative fold increase of each protein is shown (mean ± SD, n = 4–5). * *P* < 0.05 and ** *P* < 0.01.

To examine the plasma redox status in mice, we determined the activities of the antioxidative enzymes SOD and PON1, and the protein levels of PON1, apoAI, and SAA. As shown in **Fig. 4A, B**, the activities of SOD and PON1 in Lv-MsrA-GFP mice were significantly increased, and the plasma protein levels of PON1 and apoAI were significantly increased, whereas plasma SAA level was significantly reduced, compared with those in Lv-GFP mice.

### Effects of high-level hMsrA expression on hepatic gene expression and steatosis

**Fig. 5.**
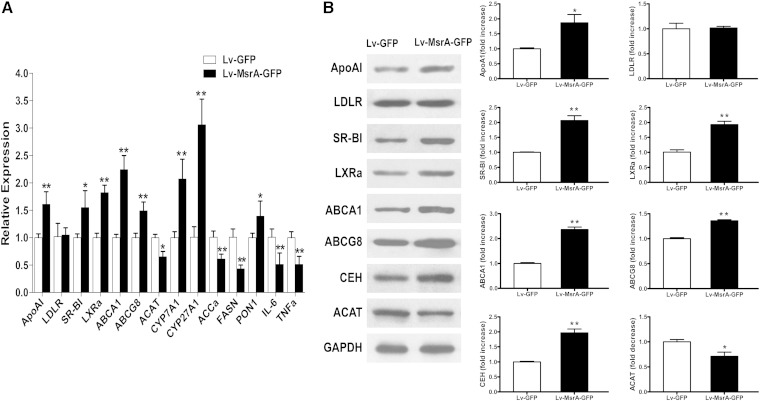
Hepatic high-level hMsrA alters expression of lipoprotein-metabolism-related or inflammation-related genes. Total RNA and proteins from the livers of Lv-MsrA-GFP and Lv-GFP mice were isolated, and gene expression levels were determined. A: Mouse apoAI, LDLR, SR-BI, LXRα, ABCA1, ABCG8, ACAT, CYP7A1, CYP27A1, ACCα, FASN, PON1, IL-6, and TNFα mRNA levels were determined by quantitative real-time PCR with the expression level of each gene normalized to the level of 18S RNA. The relative expression of each gene was shown (mean ± SD, n = 4–5). * *P* < 0.05 and ** *P* < 0.01. B: Expression of apoAI, LDLR, SR-BI, LXRα, ABCA1, ABCG8, CEH, and ACAT proteins in mouse liver were detected by Western blot analysis. Appropriate amounts of proteins were loaded and separated by SDS-PAGE, and target proteins were detected by specific primary antibodies and HRP-conjugated secondary antibodies as described in Methods. Relative fold increases of each protein are shown (mean ± SD, n = 3–5). * *P* < 0.05 and ** *P* < 0.01.

MsrA is a cellular antioxidant enzyme; the previously mentioned data indicated that hepatic expression of hMsrA ameliorated high plasma lipid levels and oxidative and inflammatory status in Western-diet-fed apoE^−/−^ mice. To investigate the underlying mechanisms, we examined hepatic expression of lipoprotein-metabolism-related genes and inflammatory factors in the Lv-MsrA-GFP mice. We found that the mRNA levels of apoAI, SR-BI, LXRα, ABCA1, ABCG8, ACAT, and bile-acid-biosynthesis-related genes CYP7A1 and CYP27A1 were increased, and the mRNA levels of ACCa and FASN, two key enzymes in fatty acid synthesis, were significantly decreased, while expression levels of LDLR were not changed (**Fig. 5A**). Changes in the expression of some of the aforementioned genes were also confirmed at protein levels. Hepatic expression of CEH and ACAT, the key enzymes in FC and CE conversion, were also measured. We found that decreased ACAT protein levels were accompanied by increased CEH protein levels in Lv-MsrA-GFP mice (Fig. 5B). Moreover, hepatic hMsrA expression significantly increased PON1 mRNA levels and decreased the mRNA levels of inflammatory factors IL-6 and TNFα in the liver (Fig. 5A).

**Fig. 6.**
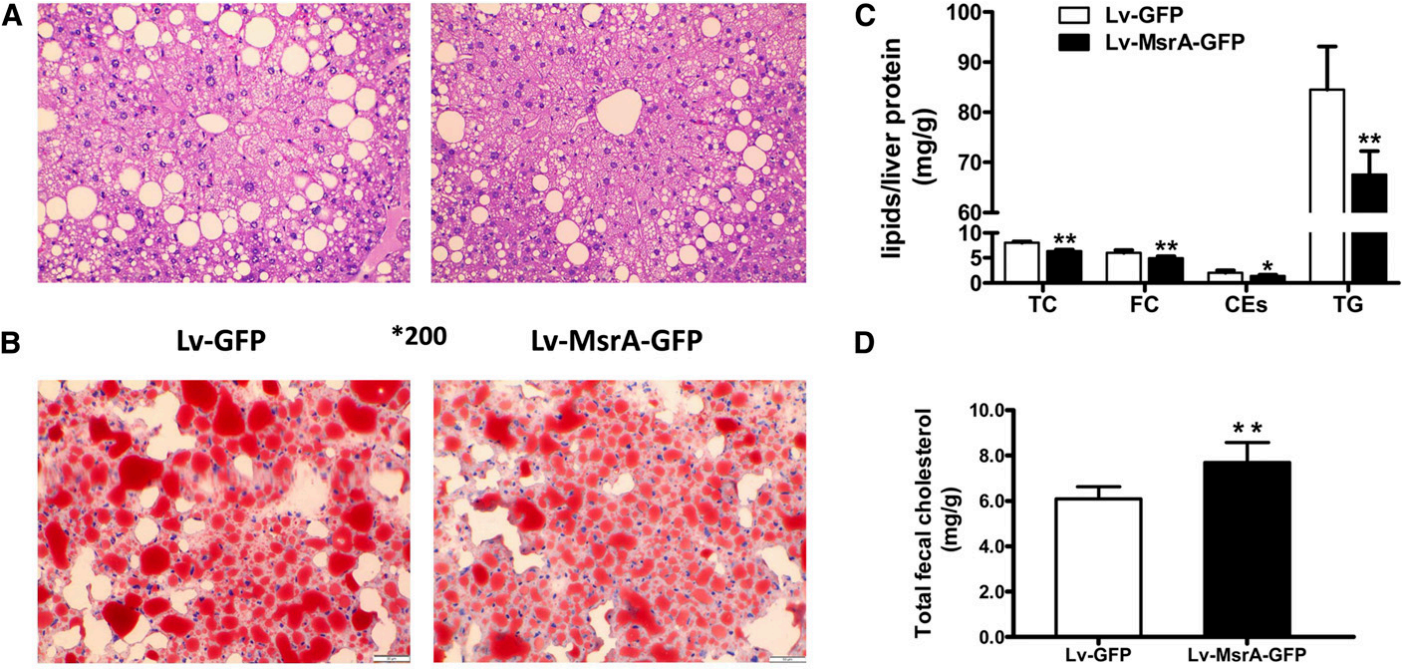
High-level hMsrA in the liver attenuates hepatic lipid content and fecal cholesterol level in apoE^−/−^ mice. Lv-GFP and Lv-MsrA-GFP mice were fed a Western diet for 12 weeks. Livers were harvested at the time of euthanasia and either used to extract total lipids or fixed for histochemical analysis. A, B: Histochemical analysis of fresh liver tissue was performed by staining with HE or Oil Red O (5 µm serial sections). C: The amounts of TC, FC, CE, and TG were determined using iospropanol as described in Methods and expressed as milligrams per gram of liver protein (mean ± SD, n = 6). D: Total fecal cholesterol was determined as described in Methods and expressed as milligram per gram of dry feces. * *P* < 0.05 and ** *P* < 0.01.

To examine the effects of hMsrA expression on hepatic steatosis in apoE^−/−^ mice, we performed HE staining and Oil Red O staining of liver slides. The results showed that lipid accumulation was significantly attenuated in Lv-MsrA-GFP mice compared with Lv-GFP mice (**Fig. 6A, B**). Consistently, when lipids in mouse liver and feces were extracted and analyzed, hepatic TC, FC, CEs, and TG levels were significantly decreased, and fecal cholesterol content was significantly increased in Lv-MsrA-GFP mice compared with Lv-GFP mice (Fig. 6C, D). These data indicated that hepatic high-level expression of hMsrA might attenuate hepatic steatosis through inhibiting fatty acid synthesis, enhancing hepatic lipid selective uptake, and increasing cholesterol excretion into the bile.

### Hepatic hMsrA expression reduced atherosclerosis

**Fig. 7.**
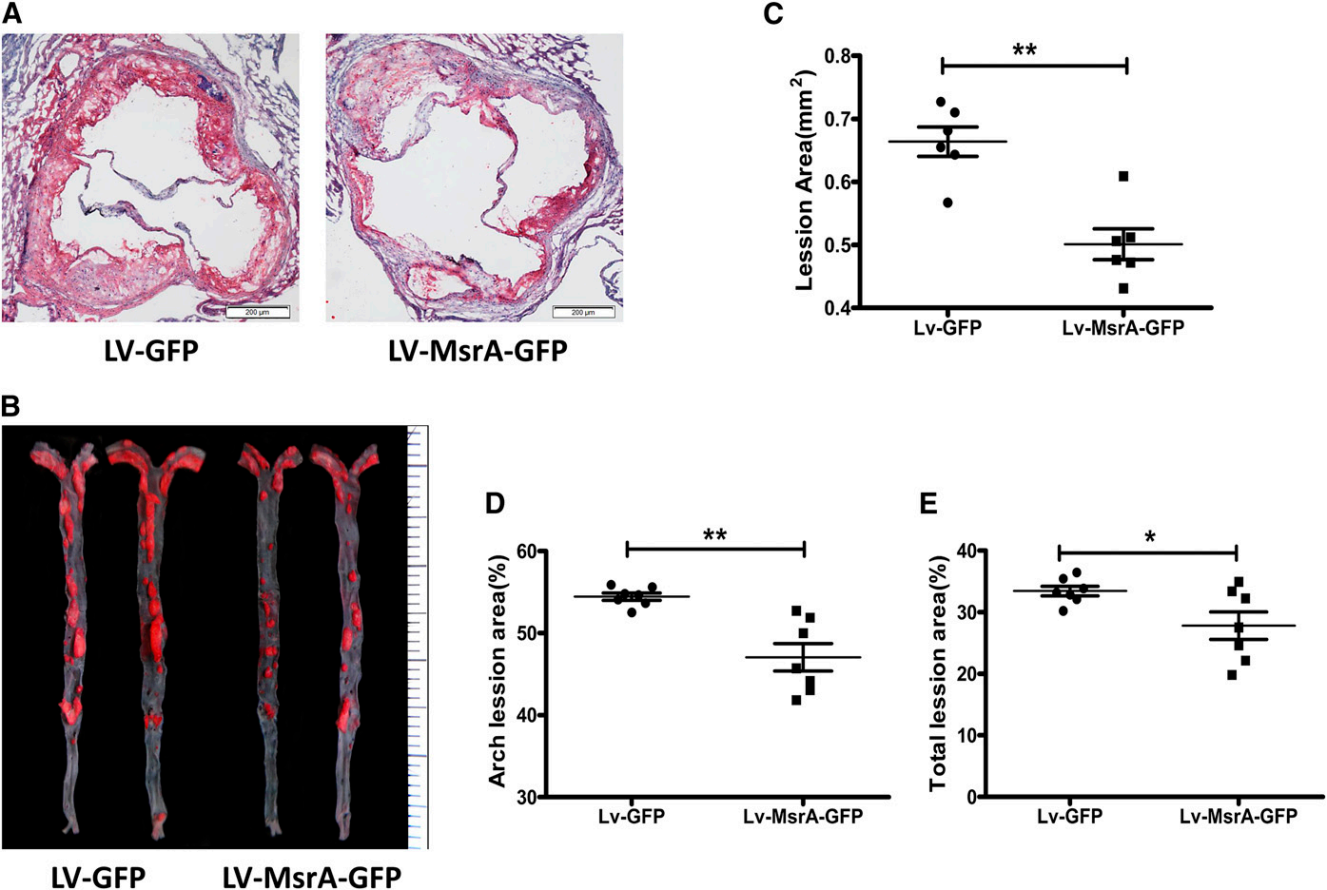
Hepatic overexpression of hMsrA reduces atherosclerosis in apoE^−/−^ mice. Lv-GFP and Lv-MsrA-GFP mice were fed a high-cholesterol Western diet for 12 weeks. Atherosclerotic lesions were examined using Oil Red O-stained cross sections of the aortic root (8 µm serial sections) and by en face analysis of the aorta and quantified by using Image-Pro Plus 6.0 software as previously described. A: Representative atherosclerotic lesions in cross sections of the aortas from Lv-GFP and Lv-MsrA-GFP mice. B: Representative en face aorta images from Lv-GFP and Lv-MsrA-GFP mice. Quantitative analysis of the cross-sectional lesion areas of the aortic roots (C), percentage lesion area in the aortic arch (D), and total lesion areas (E) was presented and analyzed by the Mann-Whitney test (* *P* < 0.05, ** *P* < 0.01, n = 6–7).

The impact of hepatic hMsrA expression on atherosclerotic lesion development in apoE^−/−^ mice was examined 14 weeks after lentiviral injection (12 weeks of Western diet feeding). Representative atherosclerotic lesions in cross sections of aortic roots and en face aorta images stained with Oil Red O are shown in **Fig. 7A, B**. Quantitative analysis of the aortic root cross sections demonstrated a significant decrease in mean lesion area in Lv-MsrA-GFP mice compared with Lv-GFP mice (0.50 ± 0.06 vs. 0.66 ± 0.06 mm^2^; *P* < 0.01; Fig. 7C). En face analysis of aortas revealed that the percentage of lesion area in Lv-MsrA-GFP mice was also significantly reduced in the aortic arch and total aorta compared with Lv-GFP mice (Fig. 7D, E). These data suggested that hepatic high-level expression of hMsrA reduced the development of atherosclerosis in apoE^−/−^ mice.

## DISCUSSION

Cellular redox status is tightly regulated by oxidant and antioxidant systems. The imbalance of these systems causes oxidative stress, which leads to dysfunctional molecules, inflammatory processes, and cell damage (19). MsrA is a powerful intracellular redox regulator that specifically reduces MetSO to Met and plays protective roles against aging and the pathogenesis of many oxidative-stress-related diseases including atherosclerosis, through the regulation of protein function and the maintenance of redox homeostasis (20). The data presented here provide the first evidence that high-level hepatic expression of hMsrA, achieved by lentiviral injection, resulted in diminished hepatic steatosis and attenuated atherosclerosis in Western-diet-fed apoE^−/−^ mice. The potential mechanisms may include amelioration of lipid metabolism dysfunction and inhibition of inflammatory status.

Liver plays a central role in lipid homeostasis, not only through lipid synthesis and apolipoprotein production, but also through the final elimination of cholesterol from the body, the important process of RCT. Homeostatic balance of lipid metabolism in the liver is essential for the prevention of hyperlipidemia, hepatic steatosis, and peripheral lipid accumulative diseases such as atherosclerosis (4, 5). It had been reported that Met-148 oxidation in apoAI could impair RCT through disrupting apoAI’s ability to activate LCAT in vitro (21); however, we did not obtain the MetSO antibodies for detecting modified apoAI according to a previous report (22). The present study showed that hMsrA overexpression in HepG2 cells significantly enhanced the expression of cellular proteins, such as LXRα, SR-BI, and ABCA1, without an effect on LDLR protein levels. In vivo results further demonstrated the effects of hepatic hMsrA expression on reducing plasma VLDL/LDL-C levels and lipid accumulation in the liver. These changes were accompanied by alterations in hepatic mRNA and protein levels of apoAI, SR-BI, ABCA1, ABCG8, and LXRα, as well as key enzymes such as CYP7A1, CYP27A1, CEH, and ACAT. These effects of hepatic MsrA may involve the modulation of the interaction between Met oxidation and cellular signaling, such as protein kinase/phosphatase-based signaling (23).

SR-BI, a cell surface high-affinity HDL receptor, plays a critical role in RCT. In the liver, SR-BI mediates cell selective uptake of CEs from spherical HDL, as well as oxidized lipoprotein. Its atheroprotective function has been demonstrated in studies using transgenic or knockout mouse models (24–26). In the present study, we showed that high-level hMsrA expression in mouse liver increased the expression of SR-BI in hepatocytes, suggesting that hepatic MsrA could assist HDL catabolism through promotion of hepatic selective cholesterol uptake.

ABC superfamily proteins are also important membrane transporters that regulate the delivery and disposal of cholesterol (27). These ABC transporters are LXRα target genes. Previous studies have indicated that ABCA1 mediates the efflux of cell cholesterol and phospholipids to lipid-poor apoAI, which is essential for the formation of nascent HDL (28). ABCG5 and ABCG8 form a heterodimer and play a critical role in the liver in the excretion of cholesterol into the bile (29). Some studies have assessed the respective roles of liver ABCA1 and ABCG8 in preventing atherosclerosis by promoting HDL biogenesis and hepatic cholesterol excretion (30, 31). Our present study suggested that hepatic MsrA may increase cholesterol movement via LXRα-mediated ABCA1 and ABCG8 upregulation, as well as apoAI synthesis and secretion. However, we did not find a difference in plasma HDL-C levels between Lv-MsrA-GFP mice and Lv-GFP mice, indicating that high-level expression of MsrA in the liver could promote HDL metabolism, including HDL-C uptake, excretion into the bile, and secretion of nascent HDL, without a net increase of plasma HDL-C levels.

Besides the increase of ABCG8 expression by MsrA, we also found that MsrA overexpression significantly increased the mRNA levels of bile-acid-biosynthesis-related genes CYP7A1 and CYP27A1 in mouse liver. Together with the finding that MsrA increases the content of TC in feces, these data support that liver overexpression of MsrA enhanced the secretion of cholesterol into bile. A limitation of our study is that we did not directly measure fecal bile acids, so we cannot exclude nonhepatic mechanisms by which hepatic MsrA may increase fecal cholesterol exit.

HDL metabolism also involves intracellular CE hydrolysis by the key cytoplasmic enzyme, neutral CEH. When HDL-C enters the hepatocytes through the SR-BI-mediated selective uptake pathway, hydrolysis of HDL-CEs is catalyzed by cytoplasm neutral CEH, and then bile acid synthesis or FC biliary secretion are promoted (32). The atheroprotective role of hepatic CEH has been previously demonstrated (33, 34). In our present study, we found that high-level expression of hMsrA in mice significantly upregulated CEH protein level in the liver. This may lead to increased hepatic hydrolysis of HDL-CEs to bridge the increased HDL-CE uptake via SR-BI and accelerated excretion into bile by the ABCG8 pathway. These synchronized processes accelerated HDL metabolism and promoted the movement of cholesterol and TG from VLDL to HDL in plasma and thus reduced plasma VLDL/LDL levels. In addition, reduced protein levels of ACAT in Lv-MsrA-GFP mice are also notable. Hepatic ACAT catalyzes cytoplasm FC esterification to generate CEs for packaging into newly synthesized VLDL (35). Therefore, reduction in hepatic VLDL production and secretion due to decreased hepatic ACAT may also contribute to the reduced VLDL/LDL levels in Lv-MsrA-GFP mice. A previous study showed that inhibition of hepatic ACAT improved hyperlipidemia (36). Furthermore, a recent study suggested that ABCA1-mediating nascent HDL biogenesis may directly induce phosphatidylinositol 3-kinase (PI3K)-mediated signaling to decrease hepatic production and secretion of TG-enriched VLDL, providing an inverse relationship between plasma HDL and TG concentrations in individuals with compromised ABCA1 function (37). We also determined the mRNA levels of lipogenic genes (ACCa and FASN) by quantitative PCR and found that hepatic expression of hMsrA significantly decreased the mRNA levels of FASN and ACCa in apoE^−/−^ mice. These data indicate that hepatic expression of hMsrA reduces TG synthesis. However, currently it is unknown why ACC and FASN were downregulated by MsrA even with increased LXRα expression. We speculate that other mechanisms mediated by MsrA may override LXRα pathway in the regulation of lipogenesis.

Although we do not exclude other potential mechanisms for hepatic MsrA to regulate lipoprotein metabolism and attenuate atherosclerosis, our data strongly suggest that MsrA can coordinately regulate *1*) hepatic lipid mobilization through increasing SR-BI-mediated cholesterol uptake and bile acid biosynthesis, *2*) CEH/ACAT-mediated FC/CEs conversion, *3*) ABCA1/ABCG8-mediated cholesterol efflux/secretion, and *4*) TG biosynthesis. These effects result in reduced plasma VLDL/LDL lipid levels, diminished hepatic steatosis, and attenuated atherosclerosis in Western-diet-fed apoE^−/−^ mice.

Dyslipidemia is often accompanied by aggravated oxidative stress and inflammation (38). Previous reports indicated that LXRα, ABCA1, and possibly SR-BI have direct effects on inflammation. LXRα is a member of the metabolic nuclear receptors; it not only serves as a key regulator in lipid metabolism and transport but also suppresses nuclear factor κB (NFκB)-mediated inflammatory signaling (39). ABCA1 may also directly act as anti-inflammatory molecule through activating the Janus kinase 2 / signal transducers and activators of transcription 3 pathway independent of its lipid transport activity (40). SR-BI has also recently been shown to suppress lipopolysaccharide-induced cytokine secretion through inhibition of NFκB activation in mice (41). Interestingly, our results demonstrated that hepatic high-level hMsrA expression in mice significantly decreased hepatic expression of inflammatory cytokines IL-6 and TNFα, as well as the plasma level of SAA, which is a marker of systemic inflammation. Moreover, plasma SOD and PON1 activities, as indicators of the antioxidant defense system in circulation, were significantly enhanced in Lv-MsrA-GFP mice. Extracellular SOD, which comes from the secreted Cu/ZnSOD and catalyzes superoxide anion conversion to hydrogen peroxide, is the main antioxidant enzyme to regulate circulating redox status (42). Our result suggests that hepatic cellular MsrA decreases oxidative stress in the liver and plasma by increasing the activity of SOD. This is consistent with a recent report suggesting that Met/Met sulfoxide modified liver homeostasis and altered the redox cellular state by increasing the activity of SOD (43). Plasma PON1, mainly located in HDL particles, predominantly reduces LDL and HDL lipid peroxidation and is considered to have the potential to protect against atherogenesis. In previous studies, we and others have demonstrated that lower plasma PON1 activity is associated with increased atherosclerosis (17, 44). The present study showed that plasma PON1 activity was increased by hepatic MsrA expression in mice, providing an additional mechanistic link by which hepatic MsrA improves HDL antioxidative and anti-inflammatory function.

In conclusion, our present study showed the first evidence that MsrA, as a specific intracellular MetSO reductase, plays an important role in lipid metabolism and redox homeostasis. We demonstrated that in apoE^−/−^ mice fed a Western diet, hepatic high-level expression of hMsrA reduces plasma VLDL/LDL, improves HDL function and redox status, and suppresses liver and plasma inflammation, resulting in diminished hepatic steatosis and attenuated atherosclerosis. We speculate that hepatic MsrA exerts its role not only through the regulation of systemic redox status but also through postoxidative stress repair of MetSO-modified proteins with direct roles in lipoprotein metabolism, such as apoAI. However, the precise molecular targets through which hepatic MsrA achieves the above functions warrant further investigation; in particular, in this study, we have not directly examined how MsrA repairs MetSO-modified proteins. Because hepatic lipoprotein metabolism and inflammation are two reciprocally regulated processes, currently we do not know if the effects of hepatic MsrA on lipoprotein metabolism or those on inflammation are the primary contributors. It will be interesting to determine the interaction between these two using our hepatic MsrA overexpression mouse model in the future.

## Supplementary Material

Supplemental Data
