# Supplementary material for: Hepatic overexpression of methionine sulfoxide reductase A reduces atherosclerosis in apolipoprotein E-deficient mice
Source: J Lipid Res. 2015 Oct;56(10):1891–900. doi: 10.1194/jlr.M058776 (PMC4583078; doi:10.1194/jlr.M058776)
Supplement: Supplemental Data [file supp_56_10_1891__index.html]

Hepatic overexpression of methionine sulfoxide reductase A reduces atherosclerosis in apoE deficient mice — Hepatic overexpression of methionine sulfoxide reductase A reduces atherosclerosis in apolipoprotein E-deficient mice — Supplemental Data 

# Hepatic overexpression of methionine sulfoxide reductase A reduces atherosclerosis in apolipoprotein E-deficient mice

## Supplemental Data

- Supplemental Tables - Table 1. Oligonucleotide primers and PCR conditions of mice genes used for quantitative real-time PCR Table 2. The information of all primary antibodies and loading amount of proteins for Western blot
- Supplemental Figs. 2 - S1: Schematic drawing of lentiviral vectors used for in vitro and in vivo study. S2: GFP expression in lentivirus-transfected HepG2 cells. S3: Hepatic high-level expression of hMsrA does not cause in vivo toxicity.
