# Supplementary material for: Hepatic overexpression of methionine sulfoxide reductase A reduces atherosclerosis in apolipoprotein E-deficient mice
Source: J Lipid Res. 2015 Oct;56(10):1891–900. doi: 10.1194/jlr.M058776 (PMC4583078; doi:10.1194/jlr.M058776)
Supplement: Supplemental Data [file supp_M058776_jlr.M058776-1.pdf]

Supplemental Data 1

Supplemental Table 1. Oligonucleotide primers and PCR conditions of mice genes used for quantitative real-time PCR

Genes	Forward primer	Reverse primer	Product (bp)	Annealing (℃)
18S	CGCGGTTCTATTTTGTTGGT	AGTCGGCATCGTTTATGGTC	219	60
ApoAI	CTCCTCCTTGGGCCAACA	TGACTAACGGTTGAACCCAGAGT	72	58
LDLR	TGTGAAAATGACTCAGACGAAC	GGAGATGCACTTGCCATCCT	87	58
SR-BI	TTTCAGCAGGATCCATCTGGTGGA	AGTTCATGGGGATCCCAGTGAC	470	60
LXRa	CTCAATGCCTGATGTTTCTCCT	TCCAACCCTATCCCTAAAGCAA	150	60
ABCA1	GGAGCTGGGAAGTCAACAAC	ACATGCTCTCTTCCCGTCAG	176	60
ABCG8	ACAAGGCTCACACAGATCTCTCA	TATAATTGGTTCCCATTCCATACTG	212	56.6
ACAT	CGCTGCGTGCTGGTCTTT	ATGCCCTTTCCTCCTCTGACA	104	56.6
CYP7A1	AAACTCCCTGTCATACCACAAAG	TTTCCATCACTTGGGTCTATGC	120	61.7
CYP27A1	GACAACCTCCTTTGTGATTG	GTGGTCTCTTATTGGGTACTTGC	166	61.7
ACCa	CGCTCAGGTCACCAAAAAGAAT	GTCCCGGCCACATAACTGAT	64	60
FASN	CCTGGATAGCATTCCGAACCT	AGCACATCTCGAAGGCTACACA	122	60
PON I	TGGTGGTAAACCATCCAGACTC	TGTGATGGTTTTCAGATGCAAG	92	60
IL-6	AGTTGCCTTCTTGGGACTGA	TCCACGATTTCCCAGAGAAC	159	60
TNFa	CGTCAGCCGATTTGCTATCT	CGGACTCCGCAAAGTCTAAG	206	60

Supplemental Table 2. The information of all primary antibodies and loading amount of proteins for Western blot

Primary antibody	Origin	Company	Cat. No.	Sample loading
MsrA polyclonal antibody	USA	Abcam	Ab16803	25 ug-50 μg
ApoAI polyclonal antibody	USA	Abcam	Ab7614	50 μg
LDLR polyclonal antibody	USA	Abcam	Ab170478	70 μg
SRBI polyclonal antibody	USA	Novus Biologicals	NB400-104	15 μg
LXRa polyclonal antibody	USA	Abcam	Ab28478	50 μg
ABCA1 monoclonal antibody	USA	Abcam	Ab18180	50 μg
ABCG8 monoclonal antibody	USA	Novus Biologicals	NBP1-71706	50 μg
ACAT polyclonal antibody	USA	Abcam	Ab93477	50 μg
CEH polyclonal antibody	USA	Abcam	Ab111544	50 μg
PON1 monoclonal antibody	USA	Abcam	Ab24261	50 μg
SAA monoclonal antibody	USA	Santa Cruz Biotechnology	SC-59680	1 μL(serum)
GAPDH polyclonal antibody	USA	Santa Cruz Biotechnology	SC-25778	
β-actin monoclonal antibody	USA	Santa Cruz Biotechnology	SC-47778	
