# Supplementary material for: Hepatic overexpression of methionine sulfoxide reductase A reduces atherosclerosis in apolipoprotein E-deficient mice
Source: J Lipid Res. 2015 Oct;56(10):1891–900. doi: 10.1194/jlr.M058776 (PMC4583078; doi:10.1194/jlr.M058776)
Supplement: Supplemental Data [file supp_M058776_jlr.M058776-2.pdf]

Supplemental Data 2

Supplemental Figure S1: Schematic drawing of lentiviral vectors used for in vitro and in vivo study. A bi-cistronic lentiviral vector PWPI containing a GFP epitope tag was used, and a 639-bp human MsrA (hMsrA) cDNA fragment was amplified by PCR from previous plasmid and subcloned into *P*ac I site in the multiple cloning regions (MCS). The final hMsrA-lentiviral construct PWPI-hMsrA-GFP was verified by DNA sequencing, and the control lentivirus was named PWPI-GFP.

Supplemental Figure S2: GFP expression in lentivirus-transfected HepG2 cells. The cells were cultured in 6 wells plate with high glucose-DMEM containing 10% FBS for 12 h, and transfected with PWPI-hMsrA-GFP vector or the control PWPI-GFP vector using Lipofectamine 2000. Three days later, cells were examined under the visible light microscope (A) and GFP expression was visualized under the fluorescence microscope (B).

Supplemental Figure S3: Hepatic high-level expression of hMsrA does not cause in vivo toxicity. Lv-MsrA-GFP or Lv-GFP mice were sacrificed at 14 weeks after lentiviral injection, body weight and spleen weight of mice were observed and plasma alanine aminotransferase (ALT) activity represented liver function was determined using Mohum's method. Data are presented as mean ± SD, n =6.

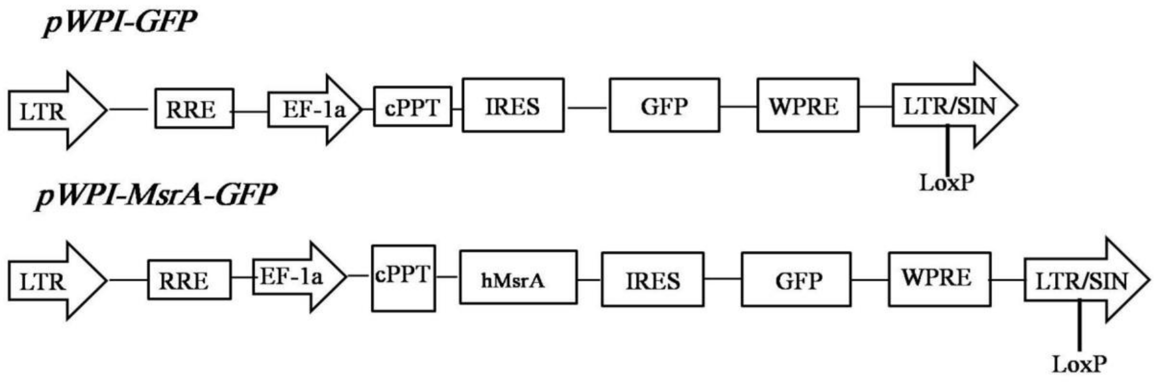

Supplemental Figure S1: Schematic drawing of lentiviral vectors used for in vitro and in vivo study. A bi-cistronic lentiviral vector PWPI containing a GFP epitope tag was used, and a 639-bp human MsrA (hMsrA) cDNA fragment was amplified by PCR from previous plasmid and subcloned into *P*ac I site in the multiple cloning regions (MCS). The final hMsrA-lentiviral construct PWPI-hMsrA-GFP was verified by DNA sequencing, and the control lentivirus was named PWPI-GFP.

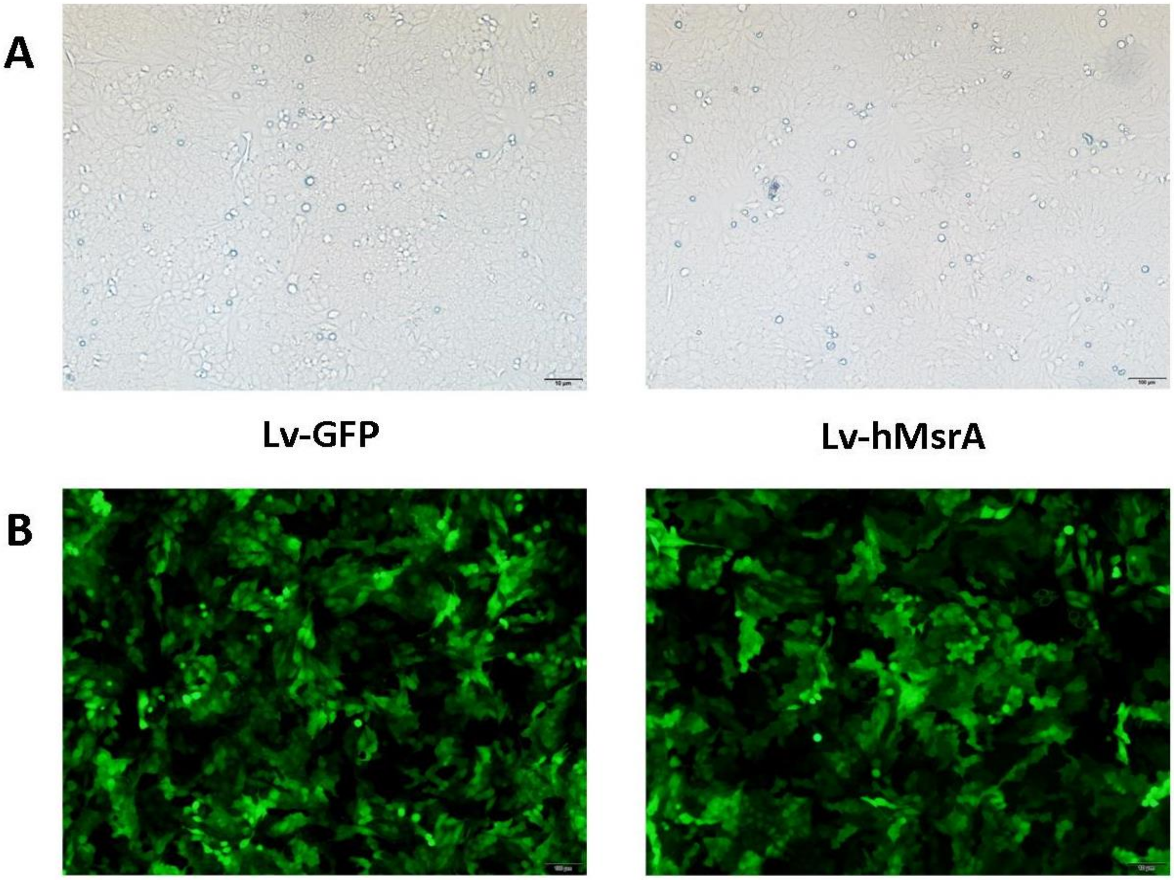

Supplemental Figure S2: GFP expression in lentivirus-transfected HepG2 cells. The cells were cultured in 6 wells plate with high glucose-DMEM containing 10% FBS for 12 h, and transfected with PWPI-hMsrA-GFP vector or the control PWPI-GFP vector using Lipofectamine 2000. Three days later, cells were examined under the visible light microscope (A) and GFP expression was visualized under the fluorescence microscope (B).

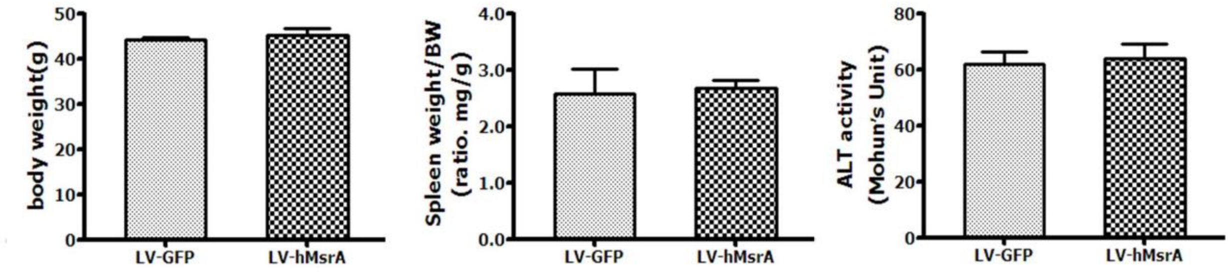

Supplemental Figure S3: Hepatic high-level expression of hMsrA does not cause in vivo toxicity. Lv-MsrA-GFP or Lv-GFP mice were sacrificed at 14 weeks after lentiviral injection, body weight and spleen weight of mice were observed and plasma alanine aminotransferase (ALT) represented liver function was determined using Mohum's method. Data are presented as mean ± SD, n =6.
